# Impact of *Janani Suraksha Yojana* on Institutional Delivery Rate and Maternal Morbidity and Mortality: An Observational Study in India

**DOI:** 10.3329/jhpn.v30i4.13416

**Published:** 2012-12

**Authors:** Sanjeev K. Gupta, Dinesh K. Pal, Rajesh Tiwari, Rajesh Garg, Ashish K. Shrivastava, Radha Sarawagi, Rajkumar Patil, Lokesh Agarwal, Prashant Gupta, Chandrakant Lahariya

**Affiliations:** ^1^Department of Community Medicine, Mahatma Gandhi Medical College & Research Institute, Cuddalore Main Road, Pillayarkuppam, Pondicherry, India; ^2^Department of Community Medicine, NSCB Medical College, Jabalpur, Madhya Pradesh, India; ^3^Department of Community Medicine, VCSG Medical Sciences & Research Institute, Srikot-Ganganali, Pauri Garhwal, Uttarakhand, India; ^4^Department of Radiology, Mahatma Gandhi Medical College & Research Institute, Cuddalore Main Road, Pillayarkuppam, Pondicherry, India; ^5^Indian Institute of Public Health, Delhi, India; ^6^Department of Community Medicine, GR Medical College, Gwalior, Madhya Pradesh, India

**Keywords:** Conditional cash transfer, Institutional deliveries, Maternal mortality, Maternal survival, India

## Abstract

The Government of India initiated a cash incentive scheme—*Janani Suraksha Yojana* (JSY)—to promote institutional deliveries with an aim to reduce maternal mortality ratio (MMR). An observational study was conducted in a tertiary-care hospital of Madhya Pradesh, India, before and after implementation of JSY, with a sample of women presenting for institutional delivery. The objectives of this study were to: (i) determine the total number of institutional deliveries before and after implementation of JSY, (ii) determine the MMR, and (iii) compare factors associated with maternal mortality and morbidity. The data were analyzed for two years before implementation of JSY (2003-2005) and compared with two years following implementation of JSY (2005-2007)**.** Overall, institutional deliveries increased by 42.6% after implementation, including those among rural, illiterate and primary-literate persons of lower socioeconomic strata. The main causes of maternal mortality were eclampsia, pre-eclampsia and severe anaemia both before and after implementation of JSY. Anaemia was the most common morbidity factor observed in this study. Among those who had institutional deliveries, there were significant increases in cases of eclampsia, pre-eclampsia, polyhydramnios, oligohydramnios, antepartum haemorrhage (APH), postpartum haemorrhage (PPH), and malaria after implementation of JSY. The scheme appeared to increase institutional delivery by at-risk mothers, which has the potential to reduce maternal morbidity and mortality, improve child survival, and ensure equity in maternal healthcare in India. The lessons from this study and other available sources should be utilized to improve the performance and implementation of JSY scheme in India.

## INTRODUCTION

The maternal morbidity and mortality have been recorded since antiquity and probably recognizing this fact, the universal declaration for human rights of 1948 in article 25 stressed that “Motherhood and childhood are entitled to special care and assistance” (1). Providentially, the maternal health issues continue to be at the forefront of global and national health policies in the last few years. The Millennium Development Goal 5 (MDG 5) calls for a three-fourth reduction in the maternal mortality ratio (MMR) by 2015 compared to 1990 levels (2-4). However, In spite all efforts, the progress in reducing maternal mortality is slow and, globally, an estimated 358,000 mothers died of pregnancy or related complications in 2008 (5). Approximately 99% (355,000) of these deaths occur in developing countries, 87% (313,000) in Africa and Asia, and more than half in 6 countries (India, Nigeria, Pakistan, Afghanistan, Ethiopia, and the Democratic Republic of Congo) only (5-6). Additionally, over 50 million pregnant women each year suffer from morbidity due to acute complications from pregnancy globally (7). The MMR in India during 2004-2006 was 254 per 100,000 livebirths with wide geographical variations, which slightly declined to 212 per 100,000 livebirths in 2007-2009 (8-10). Eight socioeconomically-backward states: Bihar, Chhattisgarh, Jharkhand, Madhya Pradesh, Orissa, Rajasthan, Uttaranchal, and Uttar Pradesh, accounted for majority of maternal deaths in India (9).

Each death or long-term complication represents an individual tragedy for the woman, her partner, her children, and her family, although a large proportion of these maternal deaths is avoidable. The main causes are known, and more than 80% of maternal deaths could be prevented or avoided through either increasing the institutional deliveries or by improving the quality of care provided to the women (3-7). Unfortunately, as late as in 2005-2006, the institutional deliveries in rural India were reported to be 28.9% (10-11). The Government of India gave high priority to promote institutional deliveries to improve maternal survival as part of national policy and also being a signatory for MDGs (4). Therefore, a well-known scheme *Janani Suraksha Yojana or* JSY was launched in April 2005 under the umbrella of National Rural Health Mission (NRHM) of India (see box) (12-13). In Hindi language, *Janani* means mother, *Suraksha* means protection, and *Yojana* means scheme. This study was planned with the objectives to assess the impact of JSY on institutional deliveries, maternal morbidity and mortality and to find out any bottlenecks in the implementation of this scheme.

## MATERIALS AND METHODS

The study was conducted in the NSCB Medical College & Hospital of Jabalpur district of Madhya Pradesh state in India (The state falls in the category of low performing states in terms of health indices in the country). This is the biggest tertiary-care hospital in this region and is the main referral centre for 8-10 adjoining districts of Madhya Pradesh. Jabalpur had a population of 1,276,853 in 2001; males formed 52% of the population, and 28.9% of the total population of the district lived in slums (14).

This observational study collected information on the number of deliveries and maternal deaths in the study hospital between August 2003 and August 2007. The maternal deaths due to abortion-related complications were excluded from the study.

**Box: *Janani Suraksha Yojana* or JSY of India (12-13, 21)**

*Janani Surkhsha Yojana* or JSY (literally meaning Maternal Protection Scheme) had been started as part of the National Rural Health Mission (NRHM) in India on 12 April 2005. The major objectives of JSY were to reduce maternal mortality ratio and infant mortality rate by encouraging institutional deliveries and focusing on institutional care among women, particularly those belonging to families below the poverty line. This is to be achieved by providing them cash at the time of delivery, along with antenatal and postnatal services. The programme divides low-performing states (LPS) and high-performing states (HPS) depending on the pre-programme level of institutional deliveries. The level of financial assistance is based on the performance level and whether the state is rural or urban. Madhya Pradesh is one of the low-performing states, and JSY was implemented in the state since August 2005.

Under this scheme, all pregnant women irrespective of age, socioeconomic status, and parity, are eligible for a cash incentive after delivery in a government or accredited private health facility in 18 high-focus states, including Madhya Pradesh, with low institutional deliveries and poor health facilities. The cash incentive is 1,000 rupees (1 US$=Rs. 50) for women from urban areas and 1,400 rupees (~$ 28) for women from rural areas. JSY is being implemented through community-level health workers (called accredited social health activists [ASHAs which means Hope in Hindi language]), who identify pregnant women and motivate them for antenatal care, institutional deliveries, and postnatal care. ASHAs receive payments of 200 rupees ($ 4) in urban areas and 600 rupees ($ 12) in rural areas per delivery assisted by them in high-focus states.

The data were collected in two phases. In the first phase, the retrospective data were collected on maternal deaths from the records of Obstetrics and Gynecology Department of the Hospital for the period from 16 August 2003 to 15 August 2005 (two years). In the second phase, all deliveries conducted in the Obstetrics and Gynecology Department of the Hospital were prospectively followed up and any maternal complications, and mortality was recorded by the first two authors since 16 August 2005 until 15 August 2007, which covers a period of two years after implementation of JSY. This design was selected to make the comparison between two time-points before and after implementation of JSY. The design has been used commonly in the past and was approved by the scientific committee of the study institute.

A standardized data-collection tool was designed, pretested, and finalized. The data were collected to include information on age, gravidity, parity, area of residence, type of antenatal care, socioeconomic status (SES), educational status, caste, and different causes of maternal mortality and morbidity. Gravidity and parity were based upon status at the time of delivery and death. Gravidity included the index pregnancy as did parity if the woman had delivered a live infant before her death. A maternal death was defined as the death of a woman while pregnant or within 42 days of termination of pregnancy, irrespective of the duration and site of the pregnancy (uterine or tubal), for any cause relating to or aggravated by the pregnancy or its mismanagement, but not for incidental or accidental causes (15). The causes of maternal deaths were divided into two categories: (i) direct obstetric causes resulting from obstetric complications, labour or puerperium, from omissions, interventions, incorrect treatment, or from a chain of events resulting from any of the above and (ii) indirect obstetric causes, including those resulting from previously existing disease or diseases that developed during pregnancy and which were not due to direct obstetric causes but were aggravated by the physiological effects of pregnancy (16). The study was approved by the ethics committee of the institution. The data were analyzed for different causes and factors affecting the maternal mortality and morbidity and to determine the sociodemographic characteristics associated with maternal morbidity and mortality. Exact matching of the data before and after implementation of JSY was done using the covariates, such as maternal age, residence (urban or rural), education, parity, socioeconomic status, and caste. The impact of JSY on maternal mortality and morbidity ratio and factors associated with these were analyzed using the Pearson chi-square test. The *t*-test was applied wherever relevant. Differences with the p values of p≤0.05 were considered statistically significant.

## RESULTS

The absolute number of institutional deliveries in the hospital during the 2 years before (2003 to 2005) and after (2005 to 2007) the implementation of JSY was 3,929 and 5,604 respectively, showing a 43% increase.

Table 1 shows the background and sociodemographic profile of the study subjects in the two periods. It is worth noting that proportionately more pregnant women younger than 19 years of age came for institutional deliveries. This age-group constituted almost 10% of the institutional deliveries conducted in this hospital during the study period. More than two-thirds of the respondents opting for institutional delivery were from rural background, and the number has increased after the implementation of JSY (p<0.000). Most women who opted for institutional delivery had at least primary-level education. There was statistically significant (p<0.001) increase in the institutional deliveries among illiterate women and those from the lower socioeconomic strata (Class V as per modified BG Prasad scale) after implementation of JSY.

This study noted more maternal deaths (total 137) during the second phase of study period (after implementation of JSY) compared to 78 deaths reported during the 2 years prior to the implementation of JSY. The overall maternal mortality ratio (MMR) in the study population increased (from 1,985 to 2,444 per 100,000 livebirths). However, subgroup analysis of these maternal deaths by area of residence found that the MMR increased (from 1,792 to 2,576 per 100,000 livebirths) among the rural women but decreased (2,456 to 1,710) among urban women (p<0.01). The majority of maternal deaths during both phases (57.7% and 70.8%) occurred in the 21-30 years age-group. More than half of the maternal deaths were among the illiterate women. There was significant decrease in maternal deaths in multigravida after implementation of JSY (Table 2).

Eclampsia, pre-eclampsia, and severe anaemia were major morbidities both before and after the implementation of JSY. However, moderate anaemia and hepatic coma were also dominant causes of maternal deaths in the post-JSY period (p<0.001) (Table 3).

An analysis of morbidities among pregnant women in this study found anaemia as the commonest cause of morbidity. There was statistically significant increase in cases of eclampsia, pre-eclampsia, polyhydramnios, oligohydramnios, antepartum haemorrhage (APH), postpartum haemorrhage (PPH), and malaria among pregnant women attending hospital for institutional delivery (Table 4).

**Table 1.** Background and sociodemographic characteristics of study subjects

Parameter	Before implementation (Phase I) (N=3,929)	After implementation (Phase II) (N=5,604)	p value*
Age (years)			
≤19	849 (21.6)	1,758 (31.1)	
>19—24	1,298 (33.0)	1,911 (34.1)	<0.001
>24—29	1,379 (35.1)	1,506 (26.8)	
>29	403 (10.2)	429 (7.6)	
Residence		
Rural	2,789 (71.0)	4,259 (76.0)	<0.001
Urban	1,140 (29.0)	1345 (24.0)	
Education			
Illiterate	211 (5.4)	519 (09.2)
Primary: 1-4 year(s)	405 (10.3)	823 (14.7)	<0.001
Middle: 5-8 years	1,409 (35.9)	1,878 (33.5)	
High school and above [9 or more years]	1,904 (48.4)	2,384 (42.6)	
Parity			
P_1_	655 (16.6)	902 (16.1)	
P_2_ to P_4_	2,538 (64.6)	3,854 (68.7)	NS
>P_4_	736 (18.7)	848 (15.1)	
SES**			
Upper class	209 (05.3)	459 (08.2)	
Upper-middle class	701 (17.8)	1,064 (19.0)	<0.001
Middle class	1,315 (33.5)	1,729 (30.9)	
Lower-middle class	1,408 (35.8)	1,632 (29.1)	
Lower class	296 (07.5)	720 (12.8)	

*p<0.05=significant

**Socioeconomic status (SES) determined by using modified B.J. Prasad classification; NS=Not significant; Figures in parentheses are percentages

**Table 2.** Factors relating to maternal death during the period 2003-2007

Particulars	Before implementation (Phase I) (n=78)	After implementation (Phase II) (n=137)	p value*
≤20	11 (14.1)	32 (23.3)	
>20-31	45 (57.7)	97 (70.8)	<0.001
>31	22 (28.2)	8 (5.83)	
Education			
Illiterate	36 (46.1)	74 (54.0)	
Primary: 1-4year(s)	18 (23.1)	27 (19.7)	NS
Middle: 5-8 years	8 (10.2)	19 (13.9)	
High school and above [9 or more years]	16 (20.4)	17 (15.5)	
Socioeconomic status [Upper and lower classes excluded]			
Upper-middle class	30 (38.46)	37 (27.0)	
Middle class	35 (44.8)	43 (31.4)	<0.001
Lower-middle class	5 (06.4)	41 (29.9)	
Gravidity status			
G_1_	28 (35.8)	71 (51.8)	
G_2_ to G_4_	41 (52.7)	63 (46.0)	
≥G	9 (11.53)	3 (2.18)	0.01

*p<0.05=significant;

NS=Not significant;

Figures in parentheses are percentages

**Table 3.** Underlying causes of maternal deaths during the study period

Cause of maternal deaths	Before implementation (Phase I) (n)=78)	After implementation (Phase II) (n=137)	p value*
Eclampsia (Antepartum/ postpartum)	35 (44.9)	49 (35.8)	NS
Pre-eclampsia	15 (19.2)	15 (10.9)	NS
Pregnancy-induced hypertension (PIH)	5 (6.4)	1 (0.79)	0.02
Severe anaemia	7 (9.0)	35 (24.8)	<0.001
Hepatic coma	2 (2.6)	13 (9.5)	<0.001
Malaria	2 (2.6)	5 (3.6)	NS
Cardiac disease	4 (5.1)	4 (2.9)	NS
Placenta previa	2 (2.6)	1 (0.7)	NS
Postpartum haemorrhage	2 (2.6)	4 (2.9)	NS
Other causes **	4 (1.3)	10 (7.1)	NS

*p<0.05=significant

**Other causes included: septic shock, malignant ovarian tumour, uterine rupture, obstructed labour, disseminated intravascular coagulation (DIC), pulimonary embolism and epilepsy;

NS=Not significant;

Figures in parentheses are percentages

**Table 4.** Distribution of maternal morbidity in the study population

Morbidity factor	Before implementation (Phase I) (n=3,929)	After implementation (Phase II) (n=5,604)	p value*
Eclampsia (Antepartum+intraprtum+postpartum)	157 (4.0)	314 (5.6)	<0.001
Pre-eclampsia	56 (1.4)	114 (2.0)	0.03
Polyhydramnios	9 (0.2)	34 (0.6)	0.007
Oligohydramnios	10 (0.2)	31 (0.6)	0.03
PPH	9 (0.22)	31 (0.55)	0.02
APH	57 (1.45)	119 (2.12)	0.02
Malaria	10 (0.25)	36 (0.64)	0.007
Viral hepatitis	21 (0.53)	43 (0.76)	NS
Anaemia (Mild+moderate+severe)	2,949 (75.1)	4,190 (74.8)	NS
PIH	144 (3.6)	214 (3.8)	NS
Heart disease	24 (0.6)	51 (0.9)	NS
Gastroenteritis	39 (1.0)	71 (1.3)	NS
Diabetes mellitus	6 (0.1)	13 (0.6)	NS
Fever	21 (0.5)	32 (0.6)	NS
UTI	9 (0.2)	21 (0.4)	NS
Candidiasis	11 (0.3)	22 (0.4)	NS
Trichomoniasis	5 (0.1)	13 (0.2)	NS
Hypothyroidism	6 (0.1)	15 (0.2)	NS
Congenital uterine anomalies	6 (0.1)	9 (0.1)	NS
Pulmonary tuberculosis	6 (0.1)	15 (0.2)	NS
Neurofibromatosis+ secondary syphilis	1 (0.02)	0	NS
Acute postpartum psychosis	10 (0.2)	15 (0.2)	NS
Acute renal failure	5 (0.1)	6 (0.1)	NS
Epilepsy	5 (0.1)	9 (0.1)	NS
TORCHS infections	0 (0.0)	3 (0.05)	NS

*p<0.05=significant;

NS=Not significant;

Figures in parentheses are percentages

## DISCUSSION

We observed that the institutional deliveries have increased after the implementation of JSY. Similar trends were observed in other studies indicating that the benefits of this scheme are being availed by a wider portion of the population (17-18). The proportion of institutional deliveries in India was around 40% in 2005-2006, which continued to increase up to 72% in 2009 (11,19). In the current study, almost 85% of the beneficiaries belonged to socially-disadvantaged class (scheduled caste, scheduled tribe, and other backward classes), which have been reported by other researchers also (20). This could be explained by the fact that a large proportion of the populations in the state of Madhya Pradesh comprise these groups (15) and also that JSY increased the hospital attendance among socially-disadvantaged classes. Madhya Pradesh is one of the low-performing states in India in terms of health indices, with a significant number of pregnancies occurring in women below 20 years of age. Pregnancy in early age poses high risk to the mother and, therefore, a significant increase in institutional deliveries among the women of this age-group can play a key role in reducing MMR and achieving MDG 5. We observed that there is an increase in institutional delivery among the illiterate and lower socioeconomic class covered by JSY, indicating that the scheme is reaching the target population which earlier preferred home deliveries due to lack of education and poverty. However, the proportion of the illiterate beneficiaries was still small compared to the total beneficiaries.

The significant increase in the number of rural pregnant women for institutional delivery can be attributed to the fact that low socioeconomic status and poverty of rural population deterred them from travelling a long distance and to spend money for delivery in the past. However, it appears that introduction of cash incentive under JSY has helped in overcoming the financial barrier and motivated them to come for institutional delivery (13,21).

The finding that there was an increase in the absolute number of maternal deaths in the post-JSY period should be interpreted with caution. First, it was not a community-based study that reflected a complete picture. Second, the rise in maternal deaths in rural areas could be due to the fact that the study centre is a tertiary-level referral health facility, has a very large catchment area involving hundreds of villages, and more complicated cases from the rural areas were being referred to this facility at the last minute for lack of more skilled and sophisticated care. Thus, more pregnant mothers with high risks were referred to this hospital, and higher number of deaths occurred as only high-risk women were attending this facility. The same arguments hold good for increase in the morbidity among the study subjects in the post-JSY period. Late identification of the high-risk pregnancy cases, poor road connectivity, and poor availability of transportation further aggravated the problem by causing delay in referral (21).

It was observed that most of the deliveries occurred in the age-group of 21-30 years, and similar finding has been reported by other authors also (22). Statistically significant (p<0.01) increase in the institutional deliveries was also observed in the age-group of 31-40 years. Many of these women were coming for the first time to a health facility for delivery and this indicates that the motivators under this scheme were able to identify these high-risk mothers and referred them timely to a higher-level service centre (23).

The higher maternal mortality among illiterate women, compared to higher-educated women, has also been reported by other author, clearly depicting a direct correlation of women's literacy with maternal mortality (24). Our observation of the higher rate of maternal deaths among middle and lower-middle socioeconomic strata, compared to upper class, has been supported by other studies (23,25). A significant increase in maternal deaths in lower-middle class and lower class could be explained by the fact that more and more families in these socioeconomic classes started opting for hospital deliveries because of increased awareness and the cash incentives given from JSY and, thus, started getting reported for mortality and morbidity.

This study noted eclampsia, pre-eclampsia, and anaemia as the most common underlying causes of maternal deaths. The high number of cases of eclampsia indicates poor antenatal care and poor and untimely referral. In this study, approximately three-fourths of all pregnant women were found to have some form of anaemia. It appears that the identification and referral of anaemia cases seem to have increased. Anaemia is a known major risk factor for maternal mortality, and high prevalence of anaemia in these women should also be seen as a reflection of the suboptimal quality of antenatal care services. However, the higher risk factors among the study subjects in post-JSY period can also be taken as an indication of effective functioning of the referral system.

### Strengths and limitations of the study

This study was done in a tertiary-level hospital with a large catchment area and gives an opportunity to analyze a significant number of deliveries for mortality and morbidity pattern. However, this is not a community-based study and, hence, it is not possible to elicit the reasons why a large section of the people still does not prefer institutional deliveries and what their opinion regarding JSY was.

### Conclusions

The JSY has increased the proportion of institutional deliveries in India, specifically among vulnerable populations. There are indications that the referral mechanism and other systems under this scheme are performing well, and it can be expected that the scheme will further increase institutional deliveries and contribute in reducing maternal mortality and bring health equity in the populations. There had been an increase in the reporting of maternal mortality and morbidity. Overall, institutional deliveries increased by 42.6% after implementation of JSY in the study institute. Anaemia was the most common morbidity factor observed in this study. There was a significant increase in cases of eclampsia, pre-eclampsia, polyhydramnios, oligohydramnios, antepartum haemorrhage (APH), postpartum haemorrhage (PPH), and malaria after implementation of JSY. The scheme appeared to increase institutional delivery, which has the potential to reduce maternal morbidity and mortality, improve child survival and ensure equity in maternal healthcare in India. The lessons from this study and other available sources should be utilized to improve the performance of this scheme in India. Moreover, the high rate of anaemia among the study women demands immediate attention during the antenatal period. The long-term financial and social investment in women's literacy would definitely add to the benefits under JSY in India.

## ACKNOWLEDGEMENTS

We thank the Department of Obstetrics & Gynecology, NSCB Medical College & Hospital, Jabalpur, India, for providing us all the valuable records and Dr. Lokesh Maran for helping in statistical analysis.
